# Effects of the linker region on the structure and function of modular GH5 cellulases

**DOI:** 10.1038/srep28504

**Published:** 2016-06-23

**Authors:** Diego M. Ruiz, Valeria R. Turowski, Mario T. Murakami

**Affiliations:** 1 Laboratório Nacional de Biociências, Centro Nacional de Pesquisa em Energia e Materiais, Campinas/SP, 13083-970, Brazil

## Abstract

The association of glycosyl hydrolases with catalytically inactive modules is a successful evolutionary strategy that is commonly used by biomass-degrading microorganisms to digest plant cell walls. The presence of accessory domains in these enzymes is associated with properties such as higher catalytic efficiency, extension of the catalytic interface and targeting of the enzyme to the proper substrate. However, the importance of the linker region in the synergistic action of the catalytic and accessory domains remains poorly understood. Thus, this study examined how the inter-domain region affects the structure and function of modular GH5 endoglucanases, by using cellulase 5A from *Bacillus subtilis* (BsCel5A) as a model. BsCel5A variants featuring linkers with different stiffnesses or sizes were designed and extensively characterized, revealing that changes in flexibility or rigidity in this region differentially affect kinetic behavior. Regarding the linker length, we found that precise inter-domain spacing is required to enable efficient hydrolysis because excessively long or short linkers were equally detrimental to catalysis. Together, these findings identify molecular and structural features that may contribute to the rational design of chimeric and multimodular glycosyl hydrolases.

Cellulose, the most abundant biopolymer on earth^1^, plays a critical role in the recycling of photosynthetically fixed carbon. This polysaccharide also has great industrial relevance, both in its distinct polymer arrangements (crystalline, semicrystalline and amorphous) and in its saccharified form, i.e., free glucose units in the context of fermentation. Because of its association with other complex polysaccharides, extracting cellulose from lignocellulosic materials requires the combined action of a broad range of carbohydrate-active enzymes (CAZymes), principally glycosyl hydrolases (GH) (EC 3.2.1.-)^2^. Of these, GH family 05 (GH5) is among the largest and most functionally diverse group; it is characterized by a canonical (β/α)_08_ fold and a broad spectrum of substrates (more than 20 experimentally verified activities on different polysaccharides). In addition, GH5-family enzymes generally contain an accessory module that is linked to the catalytic core and mediates adsorption to the substrate^3^.

Although cellulose is a chemically simple molecule (β-1,4-linked glucosyl residues), its extraction from the plant cell wall and its subsequent hydrolysis are difficult because it crosslinks with hemicelluloses and exhibits low solubility in aqueous solution. For this reason, the enzymatic depolymerization of cellulose requires at least three complementary activities: those of endoglucanases (endo-β-1,4-glucanases), cellobiohydrolases (exo-β-1,4-glucanases) and β-glucosidases. In nature, cellulose is primarily broken down through two pathways: one is widespread among aerobic bacteria and fungi and involves only individual glycosyl hydrolases, and the other is restricted to a few anaerobic microorganisms, in which these complementary enzymes form a macromolecular assembly called the cellulosome^4^.

Between these two main pathways lies an alternative strategy that involves multimodular cellulases harboring different substrate-binding modules and catalytic domains in the same polypeptide. The mechanism of this intermediate strategy was recently described by Brunecky and co-workers, who have characterized the enzyme CelA from the thermophilic bacterium *Caldicellulosiruptor bescii*. CelA contains two catalytic domains belonging to GH families 09 and 48 and three type III cellulose-binding modules. Interestingly, this enzyme displays a better saccharification potential than commercial mixtures of endo- and exoglucanases, thus highlighting the biotechnological relevance of multi-component enzymes in bioconversion processes^5^. In terms of applicability, most efforts have focused on engineering multispecific enzymes or cocktails capable of lignocellulose deconstruction independently of physicochemical pretreatment. Indeed, the heterologous expression of a GH5 endo-β-1,4-glucanase from *Acidothermus cellulolyticus*, which acts synergistically with CelA *in vitro* has been shown to enhance the extracellular cellulolytic activity of *Caldicellulosiruptor bescii* 6. The well-established importance of modular GHs, and more recently of multimodular enzymes, in biomass saccharification has enabled new biotechnological applications. However, the molecular basis for the enhanced synergistic action of these modular enzymes is not fully understood, particularly regarding the contribution of the linker region.

In general, linkers are defined as flexible regions connecting two adjacent domains within modular proteins. Currently, the available information on the molecular dynamics of linker regions is scarce, mainly because of technical limitations. In this sense, recent progress in small-angle X-ray scattering (SAXS) data collection and analyses combined with crystallography/NMR of structured domains and computational modeling have fueled early studies of the molecular characteristics of linkers^7^. Although linkers are highly divergent in their lengths and sequences, those from lignocellulose-degrading enzymes generally show a bias toward certain amino acids, such as proline, glycine, serine and threonine^8^. In fact, whereas proline residues increase linker rigidity and enable extended conformations^9^, the presence of glycine residues provides additional flexibility to allow for the proper orientation between domains^8^. Moreover, glycosylation on serine or threonine residues of fungal linkers has an important effect on protein flexibility, preventing linker collapse and stabilizing conformations with large distances between adjacent modules^9^,^10^. In addition, the glycosylation of eukaryotic linkers renders them more resistant to proteolysis and enhances their cellulose-binding affinity^11^.

Previous studies have shown that linker length also plays a fundamental role in the activity of processive cellulases, because shortening or deleting the linker reduces enzymatic activity on crystalline cellulose^12^. Linker length also appears to play a role in the thermal adaptation of some cellulases, as has been reported for Cel9A from the thermophile *Thermobifida fusca* and Cel5G from the Antarctic *Pseudoalteromonas haloplanktis*^13^,^14^. Furthermore, in this psychrophilic cellulase, the predominance of negatively charged residues and the presence of short disulfide-bridged loops leads to extended conformations of the Cel5G linker region^15^.

Despite these findings, the role of linker regions in modular non-processive GH5 cellulases has yet to be elucidated at the molecular level, particularly with respect to length and rigidity. Thus, in this work, comprehensive biochemical, mutational and biophysical analyses were carried out to determine the influence of different linkers on the structure and function of a modular GH5-CBM3 endo-β-1,4-glucanase from *Bacillus subtilis* (BsCel5A), whose structure has been elucidated by our group^16^. Collectively, the results presented here highlight the relevance of the linker region in enzyme function, revealing that a certain degree of rigidity and distance between the domains are critical for the correct function of modular GH5 cellulases. In addition, because BsCel5A has a very common modular architecture among GHs (catalytic domain tethered to a substrate-binding module) and belongs to the polyspecific GH5 family, it represents a valuable model for study. Thus, in addition to contributing to a better understanding of the role of the linker region in GH5 cellulases, our findings might be useful for the development of new chimeric and multifunctional enzymes.

## Results

### Rational design of linker-region mutations

**Figure 1:**
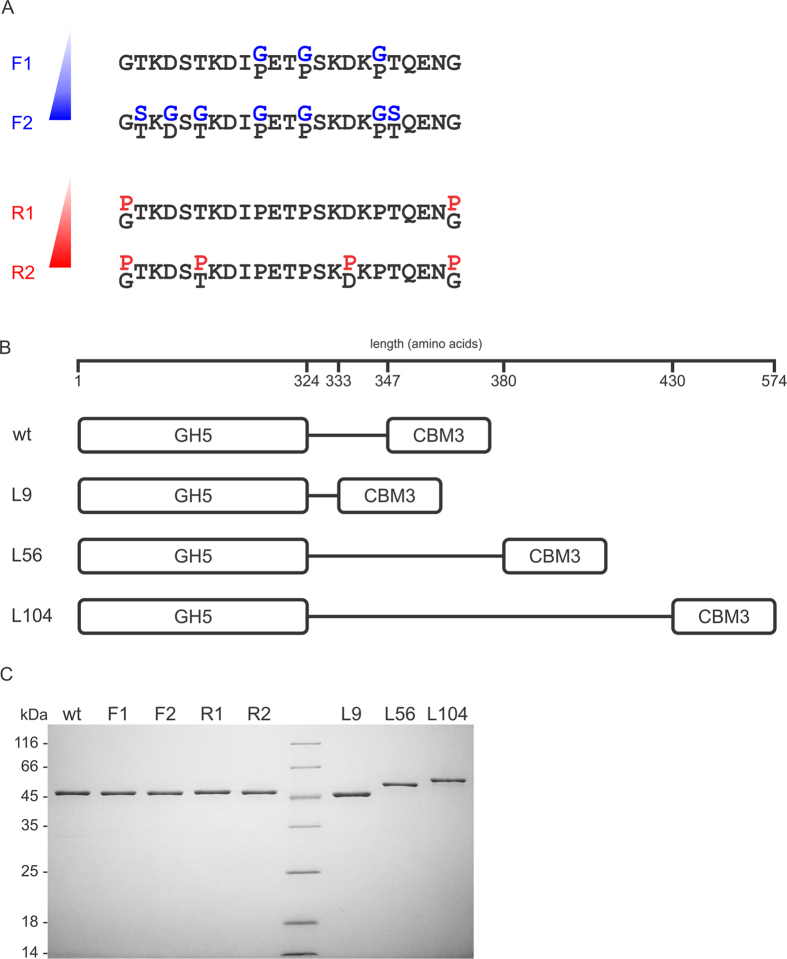
Rational design and purification of BsCel5A variants. (**A**) Sequences of linkers showing the residues that were changed to modify linker flexibility. (**B**) Representation of chimeric proteins with linkers of variable length. (**C**) SDS-PAGE analysis of the purity of wt BsCel5A and its mutants. Purified proteins (2 μg) were loaded onto a 13% polyacrylamide gel and stained with Coomassie brilliant blue G-250.

Sequences with similar domain architecture to BsCel5A were retrieved from the NCBI Entrez Protein Database, aligned and edited, thus resulting in a set of unique sequences corresponding to the inter-domain linkers, which were further clustered into seven groups on the basis of their ontogeny (Figure S1). In general, most of the linker sequences are 23 residues long and present a similar amino acid composition, including terminal glycine residues and proline residues at positions 334, 337 and 342 (based on BsCel5A numbering). The conservation of these residues suggests a putative role in enzyme function and/or structure, and hence these glycine residues were replaced with proline and vice-versa to investigate the structural and functional relevance of linker flexibility (F1 and R1, Fig. 1A). Moreover, to further enhance this feature, additional residues were substituted with glycine or proline, taking into account their natural occurrence (F2 and R2, Fig. 1A).

To examine the effects of changes in length, chimeric proteins with linkers that were two-fold (L56 – 56 residues long) and four-fold (L104 – 104 residues long) longer than that of wild-type (wt) BsCel5A were synthesized on the basis of the sequences of GH5-CBM3 cellulases from *Paenibacillus* genus (Fig. 1B and S1). In addition, a protein featuring the non-natural linker GSGSGSGSG (L9 – 09 residues long) was designed by considering the minimal distance required to prevent steric clashes between the catalytic and accessory domains (Fig. 1B) because linkers shorter than that of the wt enzyme were not observed between homologous sequences (Figure S1).

All BsCel5A variants were successfully expressed in *E. coli* in the folded and soluble form and purified by three chromatographic steps, thus yielding samples with high purity and homogeneity (Fig. 1C).

### The linker does not have a role in BsCel5A stability

As a first step, possible structural changes in BsCel5A variants were assessed by circular dichroism spectroscopy (Figure S2). When the CD spectra from the tailored proteins were compared with the spectrum of wt BsCel5A, no appreciable changes in secondary structure were observed, thus suggesting that redesigning the linker region did not affect the integrity of the structured domains. The CD spectra exhibited a characteristic minimum between 210 and 215 nm, indicating significant amount of β structures, a result consistent with the eight β-sheets in the catalytic domain and the β-sandwich fold of CBM3 observed in crystallographic and NMR structures^16^.

**Figure 2:**
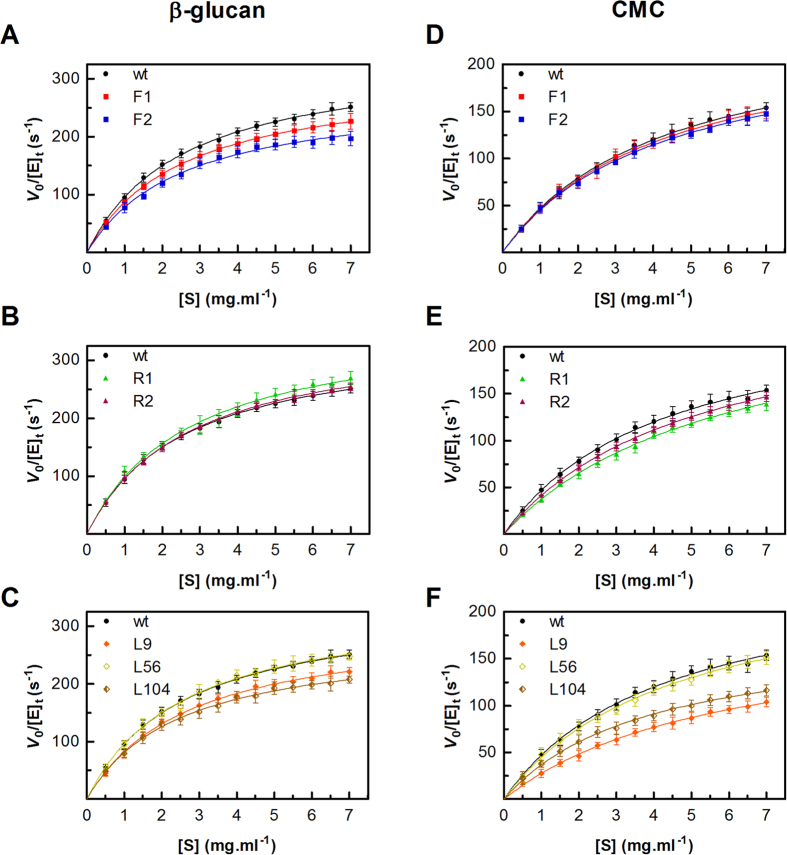
Effect of flexibility and linker extension on the kinetic behavior of BsCel5A. The initial hydrolysis rates, V_0_, were determined in triplicate from time-courses obtained at each substrate concentration for β-glucan (**A–C**) or CMC 4M (**D–F**). For purposes of comparison, the curve corresponding to the wt enzyme was included in all graphs. The results are representative of at least five independent experiments.

Furthermore, the influence of the linkers on BsCel5A stability was analyzed by thermal unfolding assays. All BsCel5A variants behaved very similarly to the wt protein under thermal denaturation, with a characteristic two-state transition curve and a melting temperature of approximately 60 °C (Figure 2S). This result may suggest a cooperative unfolding process between the two domains that is unaffected by the length or composition of the linker. The tertiary structure was also probed by differential scanning fluorimetry, and as expected, no changes were observed in thermal stability (data not shown).

These results indicate that the structure of the domains remained largely intact despite changes in the linker and that linker length and composition do not have an appreciable role in the structural stability of GH5-CBM3 cellulases.

### Linker length and flexibility are critical for enzyme performance

Because a previous study has shown that linker stiffness and length are important modulators of cellulase activity at low temperatures^14^, the effects of linker modifications on the temperature dependence of BsCel5A were evaluated. In contrast to the psychrophilic cellulase from *P. haloplanktis* (Cel5G), BsCel5A and its variants showed no changes in the thermal activation curves (Figure S3). This difference is explained by the inter-domain segment of Cel5G being stabilized by three disulfide bridges and being composed of multiple TSP_03 motifs, which are short, aspartate-rich repeats with high affinity for calcium ions.

Regarding substrate selectivity, the release of reducing sugars was not detected when each version of BsCel5A was incubated in the presence of Avicel^®^ PH-101 SIGMA for 36 h, thus indicating their inability to hydrolyze microcrystalline cellulose (data not shown). However, both the wt and mutant enzymes showed a clear preference for β-glucan over CMC, a common feature of endo-β-1,4-glucanases belonging to the GH5_02 subfamily^3^ (Figure S4).

To better understand the effect of the linker on enzyme activity, steady-state kinetics studies were performed using either β-glucan or CMC as the substrate. Regardless of the substrate, all BsCel5A versions behaved as typical Michaelis-Menten enzymes (Fig. 2). Consistently with the results of the substrate selectivity assays, the saturation curves also reflected the striking preference of BsCel5A toward β-glucan, displaying hydrolysis rates nearly two-fold higher than that for CMC (Fig. 2A,D).

Proteins with more flexible linkers had lower catalytic efficiency (*k*_cat/_*K*_M_), owing to a reduction in the turnover rate (*k*_cat_), as evidenced by comparing F2 and F1 muteins (Table 1). In contrast, the turnover rates tended to be higher in mutants with rigid linkers (Table 1). Nevertheless, regardless of the substrate, enzyme affinity decreased as a consequence of loss in degrees of freedom when the terminal glycine residues (glycine residues at the extremities of the linker region) were replaced with proline. This effect was more pronounced on CMC, thus leading to lower catalytic efficiencies (Fig. 2B,E).

Surprisingly, BsCel5A harboring a 56-residue inter-domain spacer (L56) showed wt-like behavior, in contrast to the variant with the longest linker (L104), which had a detrimental effect on the turnover rates for both substrates (Table 1). The shortest linker (L9) also negatively affected enzyme activity, as indicated by the reduced *k*_cat_ values for both substrates (Fig. 2C,F). In addition, the L9 chimera presented a lower affinity for CMC than the wt enzyme, thus highlighting the significance of the inter-domain distance for recognizing and binding of bulkier substrates.

### Effects of linkers on the molecular architecture in solution

**Figure 3:**
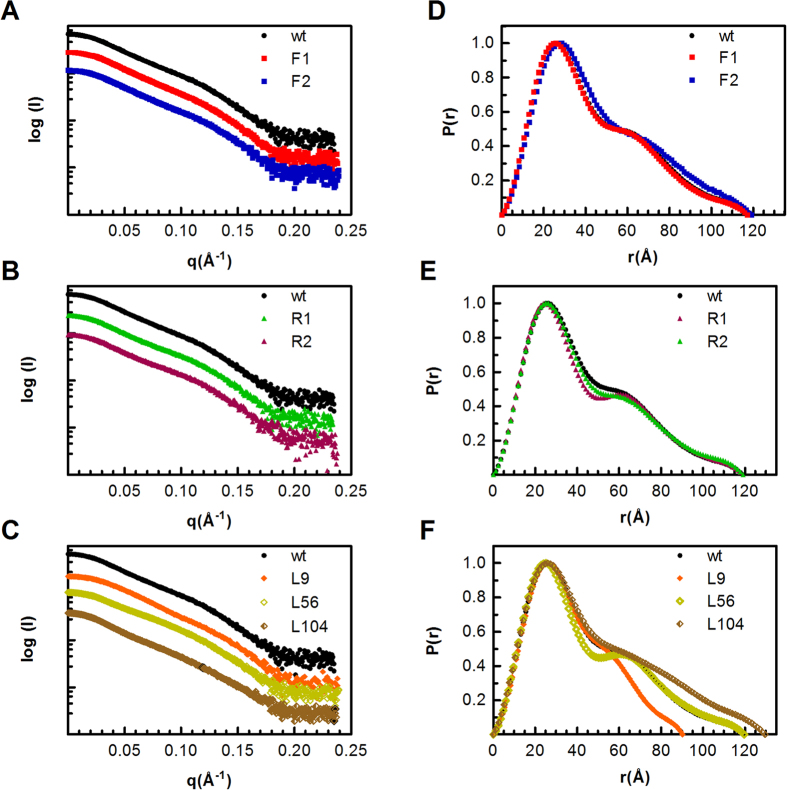
SAXS data and analyses of wt BsCel5A and its variants. SAXS experimental curves, which were arbitrarily scaled relative to each other for clarity (**A–C**), and the normalized distance distributions (**D–F**). The results are representative of three independent experiments.

To examine the possible structural changes that might explain the observed variations in the kinetic behavior of the muteins, SAXS data were collected from each BsCel5A variant. The geometric features in real space were studied on the basis of the pair-distance distribution functions P(r), obtained from the SAXS curves (Fig. 3). The scattering profiles showed that all BsCel5A proteins had dynamic conformers in solution because the curves were smoother than those expected for proteins with fixed spatial arrangements^17^. The P(r) function presented asymmetric curves with two local maxima, which correspond to the most frequently occupied inter-atomic distances that are typical for proteins composed of domains having an elongated shape in solution^18^. Notably, the P(r) profile from the L9 variant was the most symmetric, indicating a “globular” or more compact conformation. In all cases, the peak at approximately 26 Å arose from paired electron distances within the folded domains, whereas its shoulder corresponded to the distance between the catalytic and CBM3 domains. The results shown in Fig. 3 revealed that F2, L9 and L104 had greater fluctuations between modules than wt BsCel5A, whereas the R1 and L56 proteins displayed less mobile arrangements, because the smoothness degree of the secondary peak reflects the dynamic inter-domain movements^17^,^19^.

The Rg values derived from the distance distribution functions were slightly higher than those obtained by the Guinier approximation, indicating the presence of extended arrangements rather than globular architectures; nevertheless, these values were within the same range, showing the absence of aggregates and good sample quality^20^. The analysis of the P(r) distributions indicated that small changes in the amino acid composition of the linkers did not have a significant effect on the SAXS parameters, as compared with the wt protein (Table 2). However, the Rg values of the more flexible proteins F1 (Rg 34.51, D_max_ 118.2) and F2 (Rg 33.93, D_max_ 116.8) tended to be slightly smaller than that of wt (Rg 35.45, D_max_ 119.0), whereas the values for the rigid proteins R1 (Rg 35.75, D_max_ 119.5) and R2 (Rg 35.52, D_max_ 118.7) seemed to be slightly larger.

Moreover, changes in the linker length were more evident in the P(r) curves. As expected, the L9 variant exhibited a more compact arrangement with Rg and D_max_ values of 28.95 Å and 90.2 Å, respectively. In contrast, the L56 (Rg 34.86 Å, D_max_ 117.1 Å) and L104 (Rg 39.42 Å, D_max_ 129.6 Å) variants showed hydrodynamic dimensions that were smaller than expected because they have 33 and 81 additional residues in the linker regions, respectively, as compared with the wt protein. These results suggest that proteins with longer connectors, such as L56 and L104, preferentially adopt more condensed structures rather than fully unfolded structures^19^.

**Figure 4:**
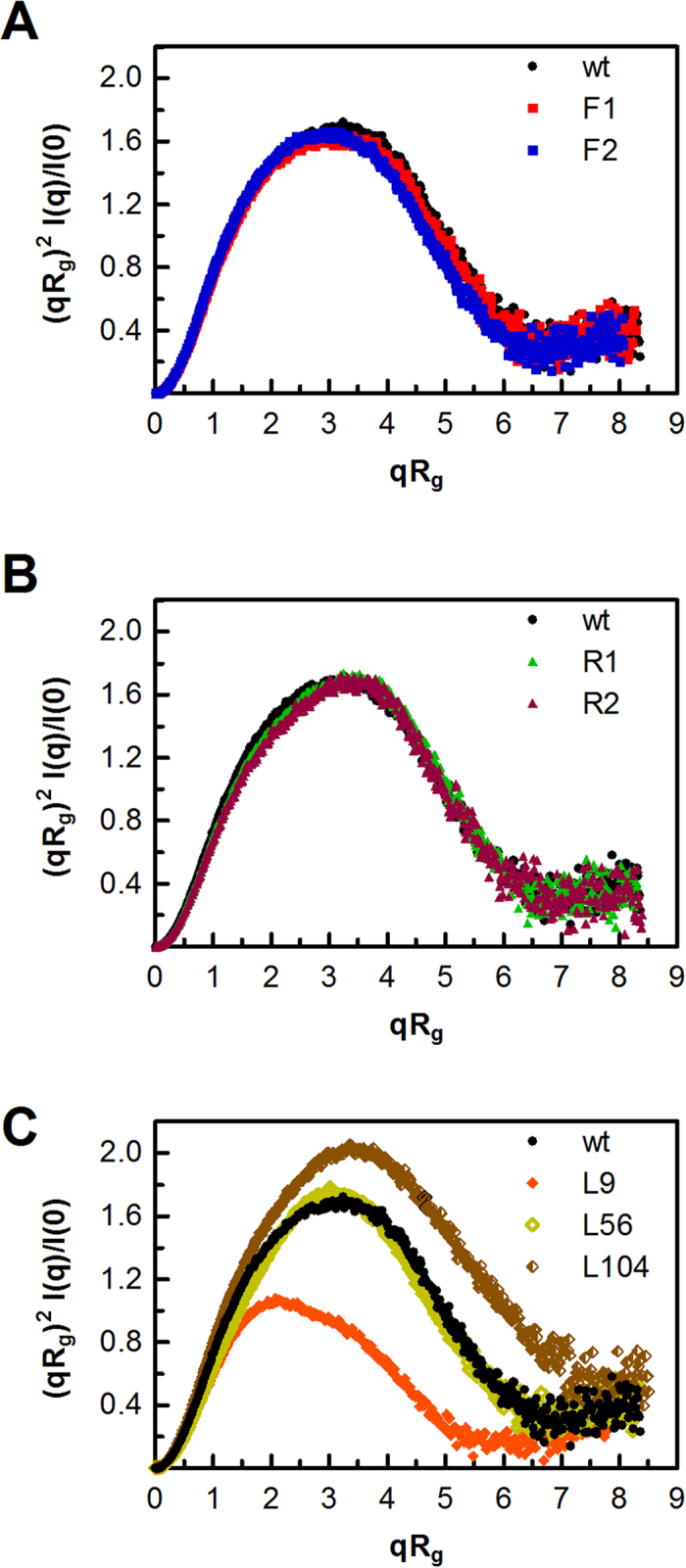
Degree of compactness from the dimensionless Kratky analysis of the BsCel5A variants, including mutants with increased flexibility (**A**) or rigidity (**B**) and chimeras with different linker lengths (**C**). The curve corresponding to the wt enzyme was included in all graphs for comparison. The results are representative of three independent experiments.

To assess compactness and global molecular flexibility, the SAXS data were analyzed by using the dimensionless Kratky transformation. At a first glance, point mutations related to linker flexibility/rigidity appeared to have negligible structural effects compared with modifications that changed the size of the linker (Fig. 4). In general, the results were similar for all versions of BsCel5A, showing a bell-like curve with a well-defined maximum and convergence towards high *q* values. Moreover, the absence of secondary peaks (typical for multimodular rigid architectures) implies that the proteins have a high degree of flexibility between their domains^17^. The smallest area under the Kratky curve was observed for L9, suggesting that this protein, which contains the shortest linker, adopts the most compact arrangement in solution (Fig. 4C). Surprisingly, the profile for L56 was similar to that of the wt protein, but the L104 variant presented the largest volume. The Kratky curves of the L9 and L104 proteins showed a slight decline at higher values of *qRg,* with a consequent rise in the baseline to a hyperbolic-like curve, which is typical for particles with an attached random coil, and suggests the presence of highly dynamic regions within the protein. As such, it is worth mentioning that Kratky curves with a single peak reflect the typical disorder of proteins composed of folded domains tethered by flexible linkers^17^,^20^.

**Figure 5:**
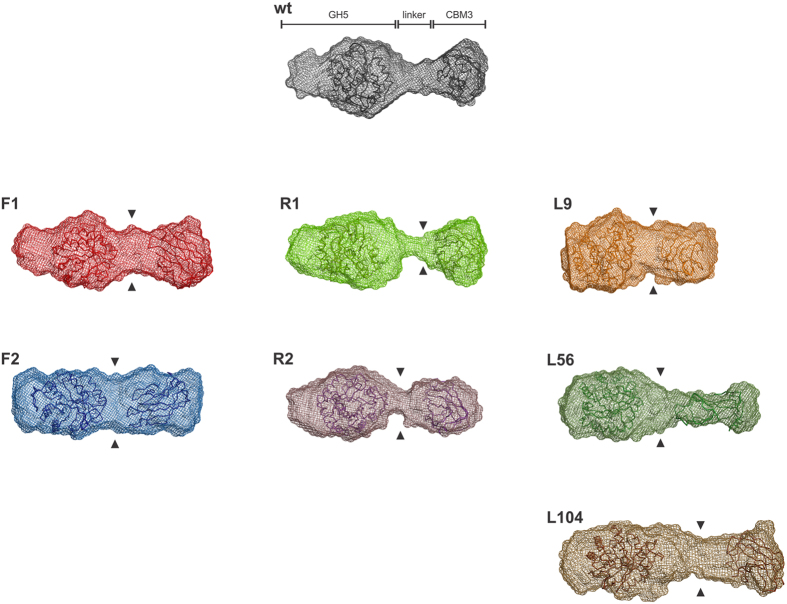
Low-resolution structural models. Average envelope of 20 independent *ab initio* models from each variant of BsCel5A. The arrowheads show the density of the linker region.

To gain further insights into the molecular shape of BsCel5A and its variants, *ab initio* low-resolution structural models were generated using DAMMIN^21^ and averaged with DAMAVER^21^,^22^. The molecular envelope of BsCel5A is characterized by an elongated shape with bi-lobular arrangements, as shown in Fig. 5, in which the bulky and globular catalytic domain (~36 kDa) corresponds to the major lobe of the model, whereas the central region is occupied by the linker (~2 kDa), and the small lobe accommodates the carbohydrate-binding domain (~16 kDa). Comparing the shapes of wt BsCel5A and its variants revealed a clear difference in the volume of the inter-lobule region (Fig. 5), which correlates with the rigidity of the linker region. In the central section, the diameter increases with flexibility, becoming almost cylindrical in F2 and thinner in the rigid mutants (R1 and R2). The molecular envelope of L9 has a shortened and continuous surface without a discretized linker region, which is in agreement with the hydrodynamic parameters for a more compact protein (Fig. 5). As suggested from the P(r) curves and Rg and D_max_ values, the L56 envelope resembles that of the wt protein, indicating a putative structural arrangement for the 33 extra residues (Fig. 5). The L104 variant showed a more extended shape with a voluminous central section, consistently with the hydrodynamic parameters (Fig. 5).

Together, these results clearly show that increases in the flexibility and size of the linker are significantly reflected in the broader electron density distributions at the central region of the SAXS envelopes, which can be attributed to the higher mobility of the flexible linkers or to the observation that longer linkers tend to adopt condensed conformations rather than fully unfolded states.

### Analysis of the inter-domain linkers by ensemble modeling

**Figure 6:**
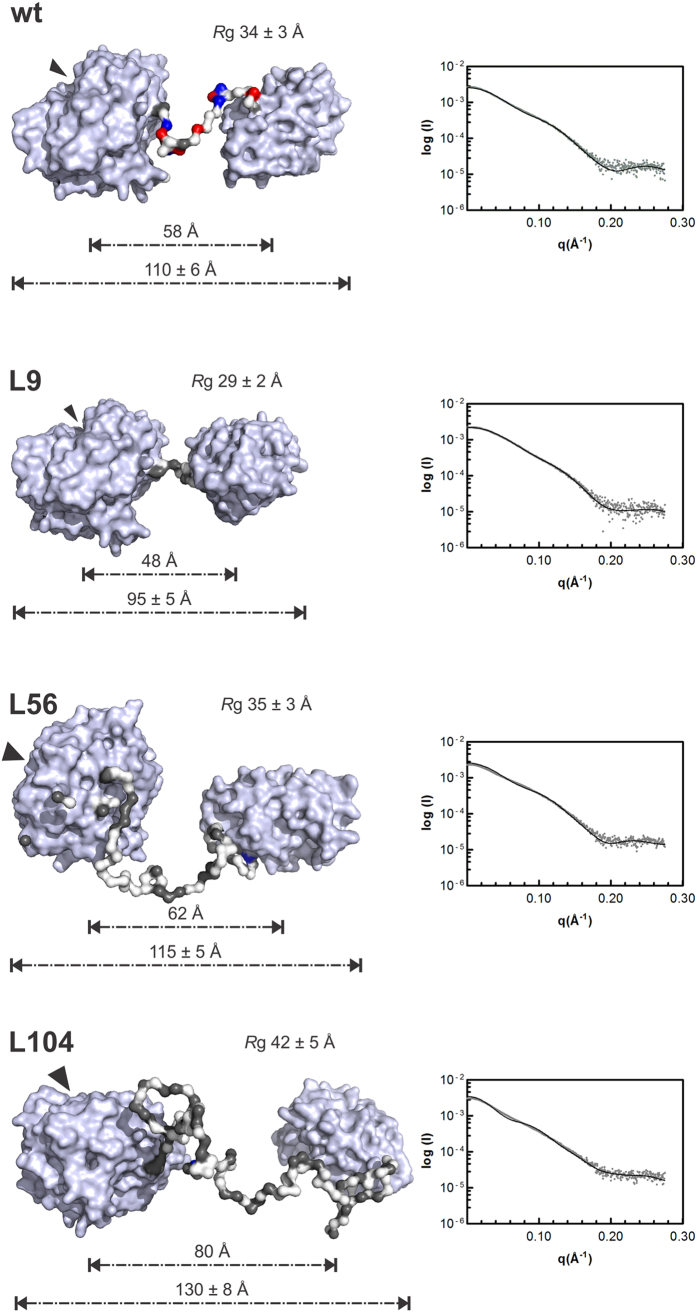
Molecular rearrangements induced by changes in linker length. Representative models of the best set of conformers from EOM (left panels). The domains are represented as gray surfaces, and the linker residues are colored according to charge: positive (blue), negative (red) or hydrophobic (gray). The black arrows indicate the location of the catalytic cleft. Fit of the experimental scattering curve (dots) and average scattering curve (line) calculated from the best models using the CRYSOL program (right panels).

To establish the best spatial distribution of conformers representing the SAXS data, 10,000 models were obtained for each protein by using the Ensemble Optimization Method (EOM). Then, the conformers that significantly adjusted to the experimental data were selected from the most representative subset (ensemble). In all cases, good fits between simulated and experimental data were obtained, giving similar Rg values but lower D_max_ values than those obtained from the P(r) distribution (Fig. 6). These differences can be explained because the D_max_ is experimentally inferred from the most extended conformations present in the solution, whereas Rg is obtained by averaging the dimensions of all conformers in solution^20^.

Proteins containing point mutations in the linker region (F1, F2, R1 and R2) had architectures similar to that of the wt protein (Figure S5). In this group, the inter-domain distance (~60 Å) coincided with that observed for the secondary peaks in the P(r) distributions (Figs 3 and S5).

Significant conformational changes were observed for muteins with linkers of variable size, on the basis of the molecular envelopes and SAXS parameters (Fig. 6). As expected, the L9 protein, which had the shortest linker, was better represented over all computed models owing to its more compact structure. Furthermore, this study also demonstrated that the L56 and L104 variants were more condensed than expected, owing to the potential structural arrangements of their linkers (Fig. 6). For this reason, the L56 variant exhibits similar Rg and D_max_ values to those of the wt protein, despite having a two-fold longer linker. From the amino acid composition (Figure S1), long linkers (L56 and L104) may be more compact as a consequence of their high ratio of hydrophobic/aliphatic residues^23^. This characteristic probably induces these segments to preferentially adopt more condensed structures rather than fully disordered and unfolded conformations.

## Discussion

In this work, the importance of the linker region in the kinetic and biophysical properties of cellulase Cel5A from *Bacillus subtilis* (BsCel5A) was investigated. This enzyme is an endo-β-1,4-glucanase (EC 3.2.1.4) belonging to the GH5_2 subfamily, with preference for β-glucans as the substrate. The limited hydrolysis rate of BsCel5A on CMC and cellulose probably indicates a topological adaptation of the active site to this carbohydrate and its nonlinear configuration promoted by the presence of mixed β linkages.

Of the 23 residues that form the BsCel5A linker, 20 are hydrophilic, and 9 are charged. Clusters of charged residues within the spacer region have been shown to increase the structural stability of class B-like penicillin-binding proteins^24^, thus suggesting that the charged residues in the BsCel5A linker may have a similar role. Recently, the ratio and distribution of charged residues have been found to act synergistically in determining the conformational properties of intrinsically disordered polyampholyte regions^25^,^26^. For example, if residues with opposite charges segregate within a sequence that follows a pattern, their electrostatic attraction may lead to chain collapse, resulting in the formation of globular or hairpins structures, but when such residues are randomly distributed, the counterbalance of repulsions and attractions yields a random coil^26^. The BsCel5A linker can be considered to be a polyampholyte because it contains stretches of residues with opposite charges, Lys^327^/Asp^328^, Lys^331^/Asp^332^ and Lys^339^/Asp^340^/Lys^341^, which becomes more evident when comparing the *in solution* conformers of wt BsCel5A and its variants (Figs 6 and S5). Thus, mutations involving these residues affect both the kinetic parameters and the hydrodynamic behavior of BsCel5A, as observed for the F2 and R2 mutants. Surprisingly, the distance distributions indicate that changes in the R2 mutein led to a less rigid linker than that observed for R1 (Fig. 3E). Therefore, disrupting the electrostatic pairs Lys^339^/Asp^340^ or Asp^340^/Lys^341^ prevents their stabilizing counterbalance, with a concomitant increase in flexibility (Fig. 5). Thus, as with glycosylation^9^, the occurrence of these local clusters of counterbalancing charges might restrict the available conformational space to ensure the correct positioning of functional domains. Moreover, the pattern of these charged amino acids is conserved in the linkers of other GH5-CBM3 cellulases (Figure S1), thus supporting their role as putative stabilizers.

The ability of enzymes to adopt numerous conformers in solution is known to be critical for catalysis^27^. Thus, although a certain degree of flexibility is necessary to allow substrate recognition and binding, excessive structural disorder can promote increased conformational heterogeneity, with a subsequent reduction in catalytic efficiency^28^. In fact, in flexible enzymes, the higher inter-domain mobility reduces their turnover rates because more time is required to sample conformers that are able to make a functional complex with the substrate^29^. Our results are in full agreement with such observations because the turnover rates slowed when the flexibility of BsCel5A increased, with F2 exhibiting the lowest *k*_cat_ followed by the L104, F1 and L9 variants (Table 1). Moreover, the highest turnover rates were achieved with the more rigid variants (R1 and R2). However, it is important to mention that some flexibility in specific regions of the linker is also necessary to allow the enzymes to adapt to different substrates because the loss of degrees of freedom at the ends (Gly^325^Pro and Gly^347^Pro) impaired the affinity of the R1 and R2 mutants toward CMC. Together, these findings emphasize the relevance of a proper and balanced distribution of rigid and flexible patches in the linker to fully exploit the catalytic properties of an enzyme.

Studies on different cellulases have suggested that the distance between the catalytic domain and the carbohydrate-binding module is essential for efficient catalysis^9^,^12^,^30^. This observation is quite relevant in processive cellulases, in which the inter-domain region directly interacts with the modules^31^. Our findings provide evidence that the inter-modular distance is also important in non-processive GH5 endoglucanases. Interestingly, although the linker in L56 is two times longer than that of the wt protein, L56 attained the highest catalytic performance among the length-chimeras of BsCel5A. Moreover, the kinetic and SAXS data provided insights into the tendency of L56 to adopt conformers with Rg values similar to those of the wt protein, revealing that spatial arrangements in which the catalytic core is maintained ~60 Å apart from the accessory domain are optimal for maximal catalytic activity. Regarding linker size, both the shorter L9 (Rg_L9_, 28.95 Å), and longer L104 (Rg_L104_, 39.42 Å) variants had a lower catalytic efficiency than did the wt enzyme (Rg_wt_ 35.45 Å), supporting the notion that the inter-modular distance and catalytic efficiency are correlated (Tables 1 and 2). Furthermore, the lower affinity of L9 for CMC also indicated that inter-domain distance is critical for the diffusion of substrates towards the active-site cleft. However, it is important to highlight that the amino acid composition also influences linker length, because a high proportion of hydrophobic residues yields less extended conformers, such as those observed in the SAXS-based ensemble modeling of the L56 and L104 muteins (Fig. 6).

Finally, the biophysical and enzymatic properties described here for the variants of BsCel5A demonstrated the relevance of the linker region in non-processive enzymes, which could be extended to other modular GH5 enzymes with different specificities. In view of the potential applicability, future experiments should be conducted in the presence of complementary cellulase activities (cellobiohydrolase and β-glucosidase) to assess the effect of different BsCel5A linkers on the hydrolysis rate(s) of insoluble cellulose(s) and/or natural substrates.

## Methods

### *In silico* analyses of the BsCel5A linker region

Protein sequences with a similar architecture to BsCel5A were identified using the CDART algorithm from NCBI Entrez Protein Database^32^. Among the 144 groups of cellulases, only one contained a GH5 catalytic core that was linked via its C terminus to a CBM3 domain. On the basis of their local alignment through Kalign^33^, the sequences were manually edited to obtain regions related to the linker region using BioEdit^34^. Finally, the sequence datasets were clustered and searched for conserved motifs using the Clustalw2-Phylogeny and MEME algorithms, respectively^35^.

### Construction of BsCel5A variants

Mutations to alter the rigidity of the linker were generated by site-directed mutagenesis using a QuikChange Site-Directed Mutagenesis kit (Stratagene, La Jolla, California, USA) and the vector pET28a(+)-BsCel5A^16^ as the template. The primers used for mutagenesis are listed in Table S1. The F1 and R1 variants were generated first by substituting proline with glycine residues at positions 334, 337 and 342 or substituting the glycines at positions 325 and 353 with proline and then were used as templates to generate the F2 and R2 mutants (see Fig. 1).

Genes encoding the chimeras containing the GH5-CBM3 domains from BsCel5A linked by peptides of variable length were synthesized into the EcoRV site of pUC57 (GenScript Co., Piscataway, New Jersey, USA). Subsequently, the coding region of each modified gene was isolated and ligated between the NheI and BamHI sites of the pET28a(+) expression vector. All variants were confirmed by sequencing the entire coding region.

### Protein expression and purification

pET28a(+) harboring the encoding sequence of BsCel5A or its variants was transformed into *E. coli* BL21(DE3) (Agilent Technologies, Santa Clara, California, USA), and the proteins were expressed for 16 h at 18 °C in selection LB medium (kanamycin) containing 0.5 mM IPTG (isopropyl β-D-thiogalactopyranoside). The cells were harvested, resuspended in lysis buffer (50 mM sodium phosphate, pH 7, 100 mM NaCl, 20 mM imidazole, 5 mM benzamidine, and 1 mM PMSF) and disrupted by ultrasound. Cell debris was removed by centrifugation (20,000 × g for 40 min), and the supernatant was filtered (0.45 μm) and loaded onto a nickel-affinity column (GE Healthcare Biosciences, Pittsburgh, Pennsylvania, USA), which was washed and eluted using a non-linear gradient of imidazole (20 to 500 mM). The fraction that eluted at 265 mM imidazole was diluted 10-fold in 50 mM sodium phosphate, pH 7, and then subjected to cation exchange chromatography (HiTrap SP HP, GE Healthcare Biosciences, Pittsburgh, Pennsylvania, USA). Finally, the fractions that eluted at 200 mM NaCl were pooled, concentrated and subsequently applied to size-exclusion chromatography (SEC) on a Superdex 75 column (GE Healthcare Biosciences, Pittsburgh, Pennsylvania, USA) that had been pre-equilibrated with 50 mM sodium phosphate buffer, pH 7, 100 mM NaCl, and 5% (*v/v*) glycerol. The sample purity was confirmed by polyacrylamide gel electrophoresis under denaturing conditions^36^.

### Enzyme assays

The enzymatic activity was determined by quantifying the amount of reducing ends released from different polysaccharides using the DNS (3,5-dinitrosalicylic acid) method and D-glucose as the standard^37^. In general, the enzyme (100 ng) was incubated for different times at 50 °C in reaction mixtures (0.1 ml) containing 200 mM Na_2_HPO_4_-citric acid buffer (pH 6), 5 mg.ml^−1^ substrate. The reactions were stopped by the addition of one volume of DNS, heated at 95 °C for 5 min, and then the products were detected by absorbance at 540 nm. Both the reaction time and the enzyme concentration were adjusted to guarantee the initial velocity conditions (i.e., linear response of product formation with respect to reaction time) to determine the kinetic parameters *K*_M_, *V*_max_ and *k*_cat_. The assays were conducted in 200 mM Na_2_HPO_4_-citric acid buffer (pH 6) at 50 °C using 1–6 mg.ml^−1^ of β-glucan from barley (Sigma-Aldrich Co., St. Louis, Missouri, USA) or CMC 4M (Megazyme Co, Bray, Wicklow, Ireland). To estimate the kinetic parameters, the experimental data were fitted to the Michaelis-Menten model using GraphPad Prism 5.0 (GraphPad Software Inc., La Jolla, California, USA). Determinations were performed in triplicate in at least three independent experiments.

### Circular dichroism spectroscopy and thermal unfolding studies

Far-UV CD spectra were obtained over the wavelength range from 195 to 260 nm using a Jasco J-810 spectropolarimeter (Jasco Analytical Instruments Inc., Easton, Maryland, USA) coupled to a Peltier temperature controller using a 1-mm quartz cuvette. The protein concentration was set to 10 μM, and the average of 10 accumulations is shown as the mean residue ellipticity (deg.cm^2^.dmol^−1^) after baseline subtraction and data normalization. To investigate the thermal stability, the CD spectra were analyzed at different temperatures ranging from 20 to 100 °C.

### Small angle X-ray scattering (SAXS)

SAXS data for all proteins were acquired on the D01A/SAXS1 beamline at the Brazilian Synchrotron Light Laboratory (LNLS, Campinas, São Paulo, Brazil). The radiation wavelength was set to 1.55 Å, and the distance between the sample and the Pilatus detector (300 k, 84 × 107 mm) was 1594.6 mm to achieve scattering *q* values of 0.01 < *q* < 0.23 Å^−1^, where *q* = (4π/λ) sin θ, and 2θ is the scattering angle. The 150-μl sample chamber was maintained at 16 °C during acquisition to minimize radiation damage. Immediately before data collection, all samples were centrifuged at 20,000 × g for 40 min at 4 °C. Scattering from the buffer was recorded before each sample measurement and subsequently subtracted. The SAXS patterns were integrated using the Fit2D software^38^, and the data were analyzed using GNOM^39^. Low-resolution envelopes of each protein were calculated from the experimental SAXS data using the *ab initio* procedure implemented in the DAMMIN program^21^. Averaged models were generated from 20 runs using the suite of programs in DAMAVER^22^. The SAXS envelopes and crystallographic models were superimposed using the SUPCOMB program^40^. Ensemble modeling was performed with the Ensemble Optimization Method (EOM), which assumes that the coexistence of different conformations of the protein in solution contributes to the experimental SAXS scattering pattern^41^. A pool of 10,000 independent models for each protein were generated using information from the high-resolution structures of the catalytic core (PDBID: 3PZT) and CBM3 (PDBID: 2L8A) of BsCel5A as constraints. The linker regions were modeled by EOM as potentially flexible regions. Once the pool of conformers was generated, a genetic algorithm was used to compare the averaged theoretical scattering intensity from independent ensembles of conformations (5–10 from the pool) against the SAXS data. The ensemble that best described the experimental SAXS data was then selected.

## Additional Information

**How to cite this article**: Ruiz, D. M. *et al*. Effects of the linker region on the structure and function of modular GH5 cellulases. *Sci. Rep.* **6**, 28504; doi: 10.1038/srep28504 (2016).

## Supplementary Material

Supplementary Information

## Figures and Tables

**Table 1:** Kinetic parameters for the hydrolysis of β-glucan and CMC.

	β-glucan			CMC		
	*k*_cat_, *s*^*−1*^	*K*_M_, mg.ml^*−1*^	*k*_cat_/*K*_M_	*k*_cat_, *s*^*−1*^	*K*_M_, mg.ml^*−1*^	*k*_cat_/*K*_M_
wt	341.37	2.55 ± 0.11	133.87	247.17	4.25 ± 0.27	58.16
F1	**307.41**	2.53 ± 0.12	121.51 (0.91)	241.09	4.24 ± 0.31	56.86 (0.98)
F2	**277.33**	2.54 ± 0.17	109.19 (0.82)	234.11	4.19 ± 0.23	55.87 (0.96)
R1	**370.14**	2.73 ± 0.16	135.58 (1.04)	257.35	**5.86 ± 0.36**	43.92 (0.76)
R2	353.11	2.69 ± 0.10	131.27 (1.03)	254.23	**5.13 ± 0.22**	49.56 (0.85)
L9	**307.90**	2.71 ± 0.15	113.62 (0.85)	**189.14**	**5.85 ± 0.49**	32.33 (0.56)
L56	344.56	2.58 ± 0.14	133.55 (1.00)	247.30	4.53 ± 0.31	54.59 (0.94)
L104	**280.61**	2.47 ± 0.16	113.61 (0.85)	**178.86**	3.86 ± 0.29	46.34 (0.80)

The numbers in bold correspond to values that were significantly different from the wt enzyme (*p* < 0.05). The relative changes compared with wild-type BsCel5A are indicated in parentheses.

**Table 2:** Overall parameters calculated from the SAXS and DLS data.

Sample	Guinier *R*_g_ (Å)	Real Space *R*_g_ (Å)	*D*_max_ (Å)	Molecular Mass^a^ (kDa)	Polydispersity (%)^b^
wt	34.0 ± 1.46	35.45 ± 0.05	119.0	54.71	11.6
F1	33.6 ± 1.64	34.51 ± 0.03	118.2	54.59	10.3
F2	33.4 ± 1.76	33.93 ± 0.05	116.8	54.46	8.8
R1	34.2 ± 1.13	35.75 ± 0.06	119.5	54.79	11.6
R2	33.6 ± 1.59	35.52 ± 0.09	118.7	54.77	10.8
L9	28.7 ± 1.26	28.95 ± 0.04	90.2	52.89	3.3
L56	33.6 ± 1.42	34.86 ± 0.03	117.1	57.13	13.2
L104	37.7 ± 1.35	39.42 ± 0.05	129.6	60.05	10.4

^a^Calculated from the protein sequence.

^b^Dynamic light scattering experiments were performed at a protein concentration of 6 mg.ml^−1^ with a DynaPro 819 at 18 °C in 50 mM sodium phosphate buffer, pH 7, 100 mM NaCl, and 5% glycerol. Polydispersity was determined with the DYNAMICS v.6.10.1.2.16 software.
