# Supplementary material for: Effects of the linker region on the structure and function of modular GH5 cellulases
Source: Sci Rep. 2016 Jun 23;6:28504. doi: 10.1038/srep28504 (PMC4917841; doi:10.1038/srep28504)
Supplement: Supplementary Information [file srep28504-s1.doc]

**SUPPLEMENTARY INFORMATION**

**Effects of the linker region on the structure and function of modular GH5 cellulases**

Diego M. Ruiz^1,a,*^, Valeria R. Turowski^1,a^ and Mario T. Murakami^1,*^

^1^Laboratório Nacional de Biociências, Centro Nacional de Pesquisa em Energia e Materiais, Campinas/SP, 13083-970, Brazil.

**Figure S1.** Analysis of the linkers from proteins with a similar architecture to BsCel5A. A) Phylogenetic tree showing seven clusters and their amino-acid frequency plots. B) Multiple sequence alignment corresponding to the GH5-CBM3 inter-domain region. The accession numbers of BsCel5A (asterisk), L56 and L104 sequences (arrowheads) are indicated on the left.

*

**B**


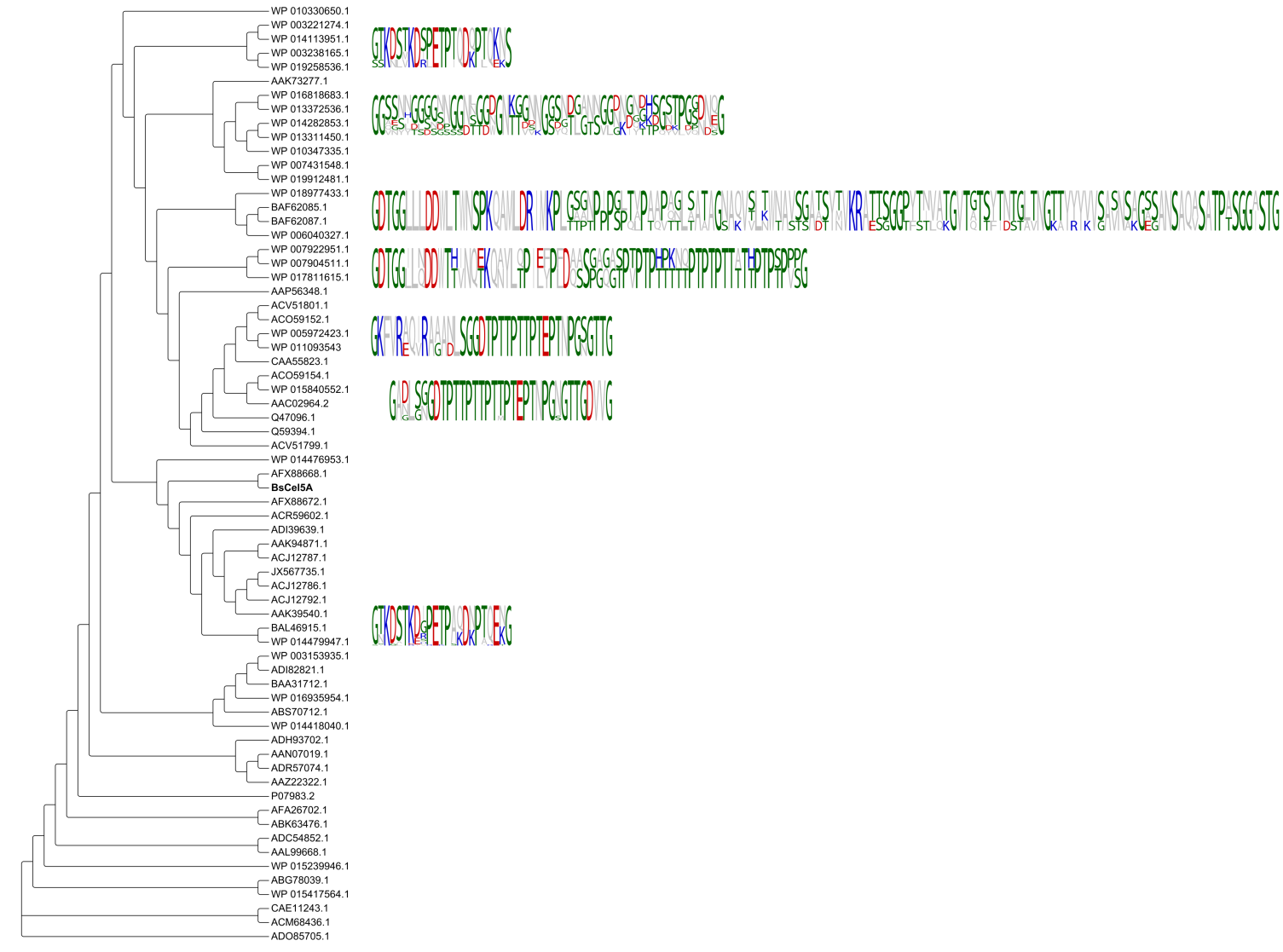


**A**

**Figure S2.** CD spectroscopy and thermal denaturation of BsCel5A and its mutants. A) Far-UV spectra (195 – 260 nm) were collected at 20°C from samples with 10 μM protein. The average spectrum of 20 accumulations is shown. B) Thermal denaturation was monitored by CD at 210 nm and 222 nm.

**Figure S3.** Influence of the linker region on temperature dependence. Cellulase activity was determined as the amount of reducing sugar released using β-glucan (A, B, C) or CMC (D, E, F) as the substrate in 50 mM Na_2_HPO_4_-citrate pH 6. All determinations were performed on triplicate aliquots. The results are representative of three independent experiments.

**Figure S4.** Substrate preference of BsCel5A and its variants. Enzyme activity was measured as the amount of reducing sugar released after 10 min of incubation under standard conditions (50 °C, 50 mM NaH_2_PO_4_ citrate pH 6, 5 mg.ml^-1^ substrate). The reactions were started by adding purified protein and stopped with one volume of DNS (3,5-dinitrosalicylic acid). β-glucan (empty bars) and CMC 4M (black bars). The determinations were performed on triplicate aliquots. The results are representative of three independent experiments.

**
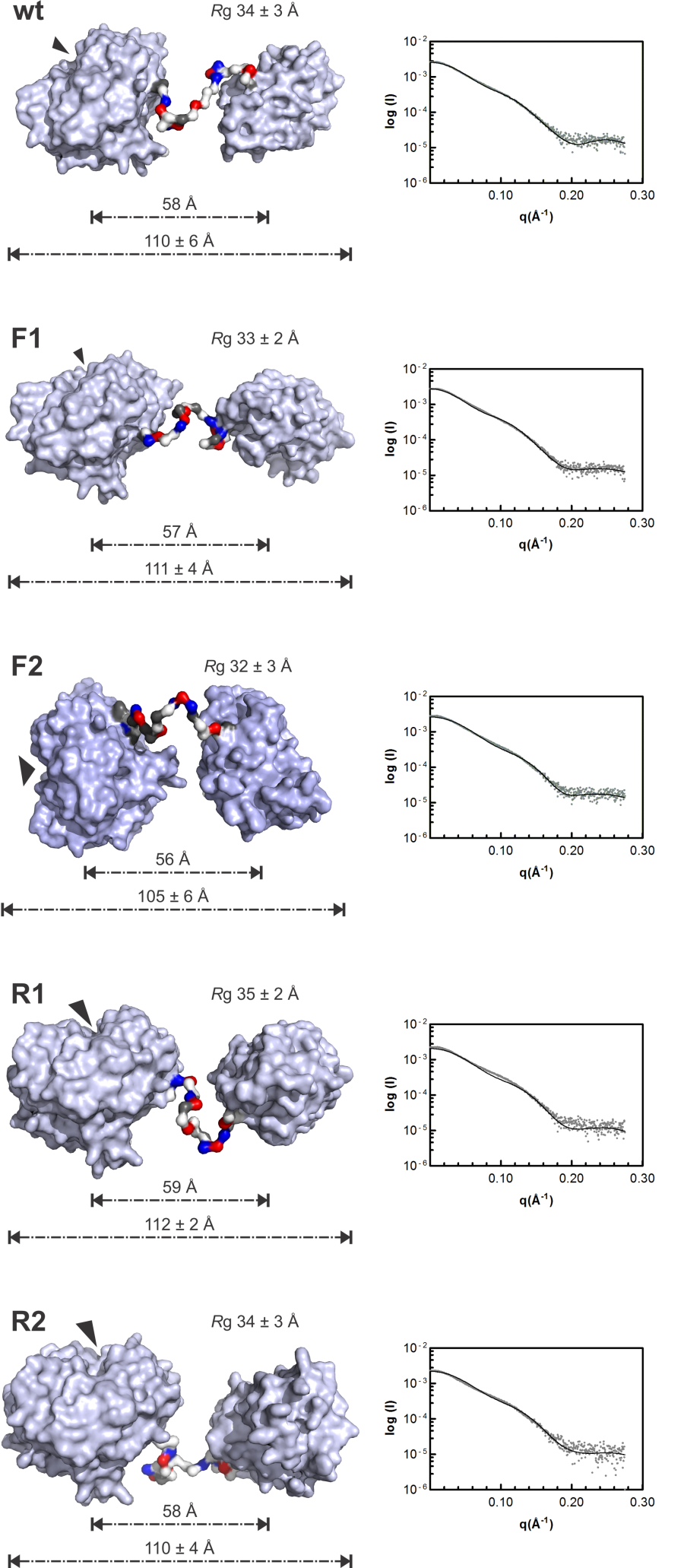
**

**Figure S5.** Comparison of the structural rearrangements induced by changes in linker flexibility. Representative models of the best set of conformers from EOM (left panels). Fit of the experimental scattering curve (dots) with the averaged scattering curve (line) calculated from the best models using the CRYSOL program (right panels).

**Table S1.** Oligonucleotides used as primers for site-directed mutagenesis.

| Primer / Direction | | Sequence | Mutation*^a^* | |  |
| --- | --- | --- | --- | --- | --- |
| Flex11 | Fw | 5´-CATTGGAGAAACGGGCTCAAAAGATAAA-3´ | P**334**G, P**337**G | | |
|  | Rv | 5´-TTTATCTTTTGAGCCCGTTTCTCCAATG-3´ |
| Flex12 | Fw | 5´-TCAAAAGATAAAGGCACACAGGAAAAT-3´ | P**342**G | | |
|  | Rv | 5´-ATTTTCCTGTGTGCCTTTATCTTTTGA-3´ |  | | |
| Flex21 | Fw | 5´-AACATTCTCGGCAGCAAAGGTTCGGGGAAGGACAGTGGAGAAA CGGGC-3´ | T**326**S, D**328**G, T**330**G, I**333**S, | | |
|  | Rv | 5´-GCCCGTTTCTCCACTGTCCTTCCCCGAACCTTTGCTGCCGAGAAT GTT-3´ |
| Flex22 | Fw | 5´-AAAGATAAAGGCTCACAGGAAAATGGT-3´ | T**343**S | | |
|  | Rv | 5´-ACCATTTTCCTGTGAGCCTTTATCTTT-3´ |  | | |
| Rigid11 | Fw | 5´-GAAAACATTCTCCCGACCAAAGATTCG-3´ | G**325**P | | |
|  | Rv | 5´-CGAATCTTTGGTCGGGAGAATGTTTTC-3´ |  | | |
| Rigid12 | Fw | 5´-ACACAGGAAAATCCGATTTCTGTACAG-3´ | G**347**P | | |
|  | Rv | 5´-CTGTACAGAAATCGGATTTTCCTGTGT-3´ |  | | |
| Rigid21 | Fw | 5´-CCGACCAAAGATTCCCCGAACGACATTCCTGA A-3´ | T**330**P | | |
|  | Rv | 5´-TTCAGGAATGTCGTTCGGGGAATCTTTGGTCG G-3´ |  | | |
| Rigid22 | Fw | 5´-GAAACGCCATCAAAACCTAAACCCACACAGG-3´ | D**340**P | | |
|  | Rv | 5´-CCTGTGTGGGTTTAGGTTTTGATGGCGTTTC-3´ |  |  | |

*^a^* The positions of each mutated amino acid is highlighted in bold.
